# Synthesis and characterization of methylammonium phosphates as crystalline approximants for anhydrous, low melting phosphate glasses†

**DOI:** 10.1039/c8ra07736c

**Published:** 2019-01-15

**Authors:** Martin Mangstl, Jan Konrad Wied, Johannes Weber, Christian Pritzel, Reinhard Trettin, Jörn Schmedt auf der Günne

**Affiliations:** Inorganic Materials Chemistry, Universität Siegen Adolf-Reichwein-Straße 2 57076 Siegen Germany gunnej@chemie.uni-siegen.de; Institute for Building and Materials Chemistry, Universität Siegen Paul-Bonatz-Straße 9-11 57076 Siegen Germany

## Abstract

Low-melting methylammonium phosphate glasses are synthesized from crystalline starting agents. To this end crystalline tris(methylammonium) cyclotriphosphate [CH_3_NH_3_]_3_P_3_O_9_, was synthesized by a novel and simple synthesis route from P_4_O_10_ and *N*-methylformamide. It, undergoes an irreversible phase transition to methylammonium *catena*-polyphosphate [CH_3_NH_3_]PO_3_. The crystal structure of the *catena*-polyphosphate was solved and refined from X-ray powder diffraction data by the Rietveld method using constraints obtained by solid-state ^31^P and ^1^H NMR spectroscopy. This compound crystallizes in a triclinic space group with *a* = 13.2236(9), *b* = 7.8924(6), *c* = 4.6553(2) Å, *α* = 91.068(4), *β* = 87.840(5) and *γ* = 106.550(3)°. Quantum chemical calculations confirm that the obtained structure lies at an energetic minimum. Finally the reaction of tris(methylammonium) cyclotriphosphate and P_4_O_10_ into methylammonium phosphate glass is presented. The synthesized, water-free phosphate glass shows a very low glass transition temperature *T*_g_ of 33 °C, which was verified by dynamic scanning calorimetry and NMR. The chain-like crystal structure of the high-temperature methylammoniumphosphate [CH_3_NH_3_]PO_3_ serves as an approximation for the short-range order of the glass.

## Abstract 2

The novel glass shows the lowest glass transition temperature known for binary phosphate glass systems without acidic protons.

## Graphical abstract

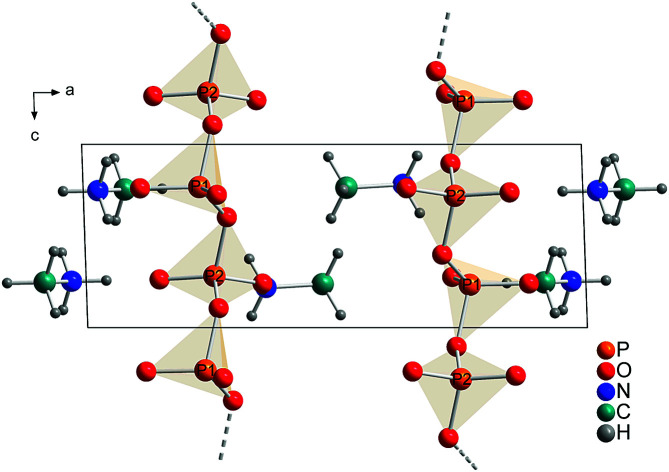

## 1. Introduction

Phosphate glasses find wide application in industry and medicine, for example as implant coatings, for tissue engineering,^1–4^ as optical materials^5,6^ and ionic-conducting materials.^7,8^ An application of glasses with low glass transition temperatures are glass seals.^9–11^ Glasses with extremely low glass transition temperatures would however open a much wider range of applications, for example enabling organic compounds as glass additives.

Lower glass transitions should be achievable for a given glass former by increasing the ionic radius of the cation of the network modifier, which lowers its cationic field strength^12^ and thus the Coulomb interaction between anion and cation. Indeed for monovalent glasses, the decrease of the glass transition temperature *T*_g_ in the sequence LiPO_3_, AgPO_3_, RbPO_3_ and CsPO_3_ is correlated with the progressive increase in the ionic radius. This effect has been attributed especially to the Coulomb interaction between the cations and the non-bridging oxygen atoms, which are responsible for the cross-links between phosphate chains.^13^ The largest stable monovalent cation in the periodic table is Cs^+^. Complex cations based on methylammonium offer an even lower cationic field strength and are the subject of this contribution.

Synthesis of crystalline methylammonium phosphates which are required as starting agents cannot proceed *via* the routine high-temperature pathway, because methyl ammonium ions decompose under these conditions. Despite this complication ammonium phosphates including mono-, di-, tri- or tetramethylammoniumphosphate find widespread application: ammonium polyphosphates are used as flame-retardant additives for organic polymers and for intumescent coatings in industry.^14,15^ In polyphosphate fertilizers usually between 50 and 75% of the phosphorus content is present in chained polymers. Only the remaining orthophosphates (monophosphates) are available for immediate uptake and the polyphosphates (phosphate rings or chains formed by condensed orthophosphates) are reduced to smaller pieces by microorganisms over time. Therefore the fertilizing effect can be warranted for a longer time period.^16,17^ In food industry ammonium polyphosphate (E545) is used for instance as additive for processed cheese due to its emulsifying properties. In contrast to the ammonium *catena*-polyphosphate II^18^ no crystal structure of methylammonium *catena*-polyphosphate is reported in literature. Solely the structures of tris(methylammonium) cyclotriphosphate^19,20^ [CH_3_NH_3_]_3_P_3_O_9_ and tris(methylammonium) hydrogenphosphate dihydrogenphosphate^21^ are known. The first had been synthesized *via* the Boullé process^22^ which requires silver salts as starting material. A larger version of the ammonium ion is the tetrasubstituted tetramethylammonium ion [N(CH_3_)_4_]^+^, for which several phosphate phases^23–25^ and phase transitions^26,27^ between them have been observed. Methylammonium hydrogenphosphate (254.2 °C) and methylammonium formate (162.1 °C) have low decomposition temperatures.^28^ Thus for their synthesis in general low synthesis temperature are required, for example making use of solvents like dimethyl sulfoxide^23,29^ or water.

In this contribution the smaller but asymmetric methylammonium ion [CH_3_NH_3_]^+^ is explored as an alternative to the tetramethylammonium ion to produce low melting phosphate glasses. Their synthesis requires starting materials of high purity. To this end a cheaper route for crystalline, water-free, non-acidic methylammonium phosphates is sought. In this context the question, if *N*-methylformamide may act as source of the methylammonium ion in the synthesis, is tested.^30^

## 2. Experimental details

### 2.1 Sample preparation

All solid reagents were stored inside a glove box (MBraun, Garching, Germany) filled with dry argon. For synthesis of crystalline trismethylammonium cyclotriphosphate 7 mL *N*-methylformamide (Alfa Aesar, 99%) was added drop-wise under ice cooling to 1.0 mmol (284 mg) P_4_O_10_ (Sigma Aldrich, 99%). After reaching room temperature the solution was heated to 45 °C for 96 hours. The obtained product was precipitated and washed five times with acetonitrile (Chemsolute, 99.9%). In order to obtain crystalline methylammonium *catena*-polyphosphate 0.6 mmol (200 mg) trismethylammonium cyclotriphosphate were heated to 245(5) °C for 2 h inside a Teflon crucible within a Schlenk flask under vacuum and subsequently cooled down slowly (2 K min^−1^).

For the synthesis of glassy methylammonium phosphate trismethylammonium cyclotriphosphate and P_4_O_10_ with different ratios were heated to 245(5) °C inside a Teflon crucible within a Schlenk flask under vacuum. After holding the temperature for 2 h the sample was cooled down fast by water quenching.

### 2.2 XRD measurements and refinements

Powder X-ray diffraction patterns were recorded at 298 K on a STOE Stadi P powder diffractometer (STOE, Darmstadt, Germany) in Debye–Scherrer geometry (capillary inner diameter: 0.48 mm) by using Ge(111)-monochromated CuK_α1_ radiation (154.0593 pm) and a position-sensitive detector. Extraction of the peak positions and pattern indexing were carried out by using FOX package.^31^ For methylammonium *catena*-polyphosphate indexing by using a Le Bail extraction with a least-squares optimization yielded a triclinic unit cell with the best score for space group *P*1̄ with *a* = 13.215 *b* = 7.887, *c* = 4.654 Å, *α* = 91.100, *β* = 87.899 and *γ* = 106.557°. All the likely space groups are subjected to a “multiple world simulation” within the FOX program (best 10 scores are shown in Table S1†). Structure solution was done with the method “parallel tempering”. The molecules were restrained in different ways: *catena*-polyphosphate units with the flexibility model “automatic from restraints, strict” and methylammonium units with the flexibility model “rigid bodies”. The molecules chosen reflect the prior knowledge concerning the NMR experiments. Rietveld refinement of the final structure model was realized by applying the fundamental parameter approach implemented in TOPAS (direct convolution of source emission profiles, axial instrument contributions, crystallite size and micro-strain effects).^32,33^

**Fig. 1:**
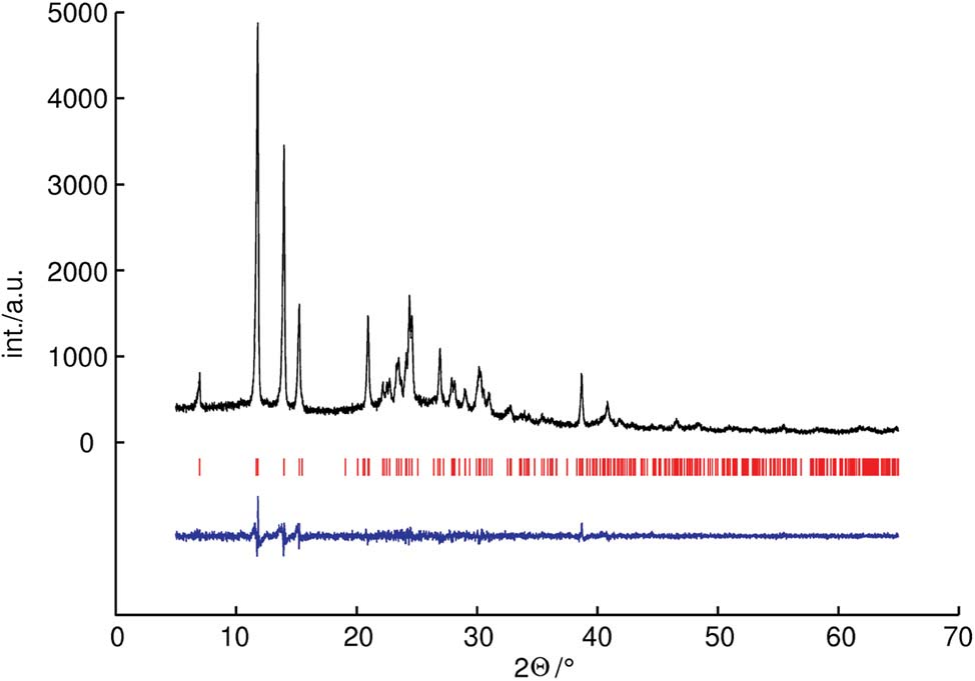
Observed powder diffraction pattern (black line) of methylammonium *catena*-polyphosphate [CH_3_NH_3_]PO_3_ measured with CuK_α1_ radiation (154.0596 pm), as well as the difference profile (blue line) of the Rietveld refinement. Peak positions are marked by vertical red lines.

It is difficult to determine the hydrogen positions by powder X-ray diffraction because of the low scattering power of hydrogen atoms. Therefore the hydrogen positions were constrained based on neutron diffraction analysis data of a known methylammonium salt. For the methylammonium cation the bond lengths of C–H were constrained to 0.96 Å (as proposed by Sheldrick) and N–C–H angles to 109.6°, the bond lengths of N–H were constrained to 0.89 Å and C–N–H angles to 109.6°.^21^ For P–O distances soft restraints were used on the basis of an average values of known *catena*-polyphosphates (1.60 Å for bridging and 1.48 Å for terminal P–O distances).^34,35^ For C–N distances soft restraints were used on the basis of the crystal structure of methylammonium chloride (1.47 Å).^36^ The crystallographic data and further details of the data collection are given in Table 1. The experimental powder diffraction pattern, the difference profile of the Rietveld refinement and peak positions are shown in Fig. 1.

**Table 1:** Crystallographic data^a^ for methylammonium *catena*-polyphosphate

**Crystal structure data**	
Formula	C_2_H_12_N_2_O_6_P_2_
Formula mass/(g mol^−1^)	222.075
Crystal system	Triclinic
Space group	*P*1̄
*a*/Å	13.2236(9)
*b*/Å	7.8924(6)
*c*/Å	4.6553(2)
*α*/°	91.068(4)
*β*/°	87.840(5)
*γ*/°	106.550(3)
Cell volume/Å^3^	465.38(5)
*Z*	2
*ρ*/(g cm^−3^) calc. from XRD	1.5848(2)
**Data collection**	
Diffractometer	Stoe Stadi P
Radiation, monochromator	CuK_α1_, *λ* = 154.06 pm, Ge(111)
Detector, internal step width/°	Linear PSD (Δ(2*θ*) = 5°), 0.01
Temperature/K	294(2)
2*θ* range/°	5.00–64.99
Step width/°	0.01
Points	6000
Number of observed reflections	342
**Structure refinement**	
Structure refinement method	Fundamental parameter model^33^
Program used	TOPAS-Academic 4.1
Background function/parameters shifted	Chebyshev/16
Number of atomic parameters	42
Number of profile and other parameters	16
Constraints/restraints	46/10
*χ* ^2^	1.191
*R*_p_	0.049
w*R*_p_	0.063

a Estimated standard deviations are given in parentheses.

### 2.3 NMR measurements

For all solid-state NMR measurements the ^1^H resonance of 1% Si(CH_3_)_4_ in CDCl_3_ served as an external secondary reference using the *Ξ* values for ^31^P as reported by the IUPAC.^37^ All experiments used a saturation pulse comb in front of every repetition delay.

The ^1^H and ^31^P solid-state NMR spectra were measured on a Bruker Avance II spectrometer operating at the frequencies of 300.13 and 121.49 MHz, respectively (magnetic flux density *B*_0_ = 7.05 T). Magic angle sample spinning (MAS) was carried out with a McKay 4.0 mm MAS probe. The ^31^P–^31^P 2D double-quantum (DQ) single-quantum (SQ) correlation MAS NMR spectrum of trismethylammonium cyclotriphosphate was obtained at a sample spinning frequency of 12.5 kHz with a repetition delay of 36 s using a transient adapted PostC7 sequence^38,39^ with a conversion period of 0.64 ms and rotor-synchronized data sampling of the indirect dimension. It accumulated 32 transients per FID. Proton decoupling was implemented using CW decoupling with a nutation frequency of 100 kHz. The ^31^P–^31^P 2D double-quantum (DQ) single-quantum (SQ) correlation MAS NMR spectrum of methylammonium *catena*-polyphosphate was obtained at a sample spinning frequency of 12.5 kHz with a repetition delay of 16 s using a transient adapted PostC7 sequence with a conversion period of 1.28 ms and rotor-synchronized data sampling of the indirect dimension. It accumulated 32 transients per FID. The ^31^P MAS NMR spectrum of amorphous methylammonium phosphate was received at a sample spinning frequency of 12.5 kHz with a repetition delay of 32 s. The ^31^P–^31^P 2D double-quantum (DQ) single-quantum (SQ) correlation MAS NMR spectrum of amorphous methylammonium phosphate was acquired at a sample spinning frequency of 12.5 kHz with a repetition delay of 20 s using a transient adapted PostC7 sequence with a conversion period of 0.96 ms and rotor-synchronized data sampling of the indirect dimension. It accumulated 128 transients per FID. The variable temperature static ^31^P NMR spectra of amorphous methylammonium phosphate were measured between 273 and 383 K with a repetition delay of 24 s. Liquid state ^1^H and ^13^C measurements were carried out on a Jeol ECZ operating at the frequencies of 500.13 and 125.76 MHz, respectively (magnetic flux density *B*_0_ = 11.75 T).

### 2.4 Differential scanning calorimetry

Differential scanning calorimetry measurements were done on a Netzsch DSC 204 F1 Phoenix calorimeter (Netzsch-Gerätebau GmbH, Selb, Germany). For the glassy methylammonium phosphate 10.9 mg of the sample were sealed within an aluminum crucible inside a glove box under argon atmosphere. The measurements were carried out under nitrogen atmosphere (20 mL min^−1^) with a heating and cooling rate of 5 K min^−1^. For the determination of specific heat capacities *C*_P_ (DIN 51007) sapphire was used as a standard.^40^

### 2.5 Computational chemistry

The atomic positions of the Rietveld refined unit cell of methylammonium *catena*-polyphosphate were relaxed under periodic boundary conditions by the Quantum ESPRESSO v.6.2 software.^41,42^ The input file for PWscf featured the usage of an energy cutoff of 80 Ry (1088 eV), and a Monkhorst–Pack^43^ like *k*-point mesh of 5 × 5 × 5 over the irreducible Brillouin zone, resulting in 63 *k*-points including the gamma point. All fractional atomic coordinates were allowed to relax freely without symmetry restrictions. Norm-conserving Troullier–Martins type^44^ pseudo potentials with PAW reconstruction^45^ (X.pbe-tm-new-gipaw-dc.UPF files, X = P, O, N, C, H) created by D. Ceresoli between 14 Sep 2009 and 25 May 2010 (ref. 46) were chosen, as we liked to calculate also NMR parameters. The PBE^47,48^ density functional was used, together with a nonempirical van der Waals correction term (VdW-DF^49–52^). The convergence threshold for self-consistency of the electronic wave function was set to 10^−13^ a.u., while the thresholds for the total energy and the atomic forces were set to 10^−12^ a.u. and 10^−9^ a.u., respectively. The cif2cell^53^ program was used to assist the input file generation. To obtain NMR parameters GIPAW calculations^54,55^ with standard setup (job = nmr, q_gipaw = 0.01, and spline_ps = .true.) were performed at the unrelaxed as well as at the relaxed structure. It turned out that the errors for calculated NMR parameters are too big for an unambiguous assignment of the phosphorus atoms.

## 3. Results and discussion

In order to obtain phase pure starting materials for the glass synthesis we established a novel synthesis route for tris(methylammonium) cyclotriphosphate (see tentative reaction equation below). Subsequently its thermodynamical stable high temperature phase methylammonium *catena*-polyphosphate was characterized. Ultimately we present the synthesis and investigation of the low melting methylammonium phosphate glass.

The reaction of *N*-methylformamide and P_4_O_10_ yielded a pale yellow powder which could be indexed within a monoclinic unit cell *P*2_1_/*n*. The powder XRD pattern is in agreement with that of tris(methylammonium) cyclotriphosphate [CH_3_NH_3_]_3_ P_3_O_9_.^19^

Solution NMR spectra of *N*-methylformamide and P_4_O_10_ after the reaction show additional signals compared to the spectra for pure *N*-methylformamide. The ^1^H NMR signal at 8.3 ppm can be assigned to the formate anion and the signal at 2.2 ppm to the methylammonium cation. Furthermore the ^13^C signal at 165.8 ppm can be assigned to the formate anion and the signal at 24.5 ppm to the methylammonium cation.^30,56^ Additionally, the formation of carbon monoxide could be confirmed by using an electrochemical sensor (see ESI†). Thus, the total reaction for the synthesis of tris(methylammonium) cyclotriphosphate could be described by the following tentative reaction equation:12CH_3_NHCOH + 6H_2_O + 3P_4_O_10_ → 4[CH_3_NH_3_]_3_ P_3_O_9_ + 12CO

**Fig. 2:**
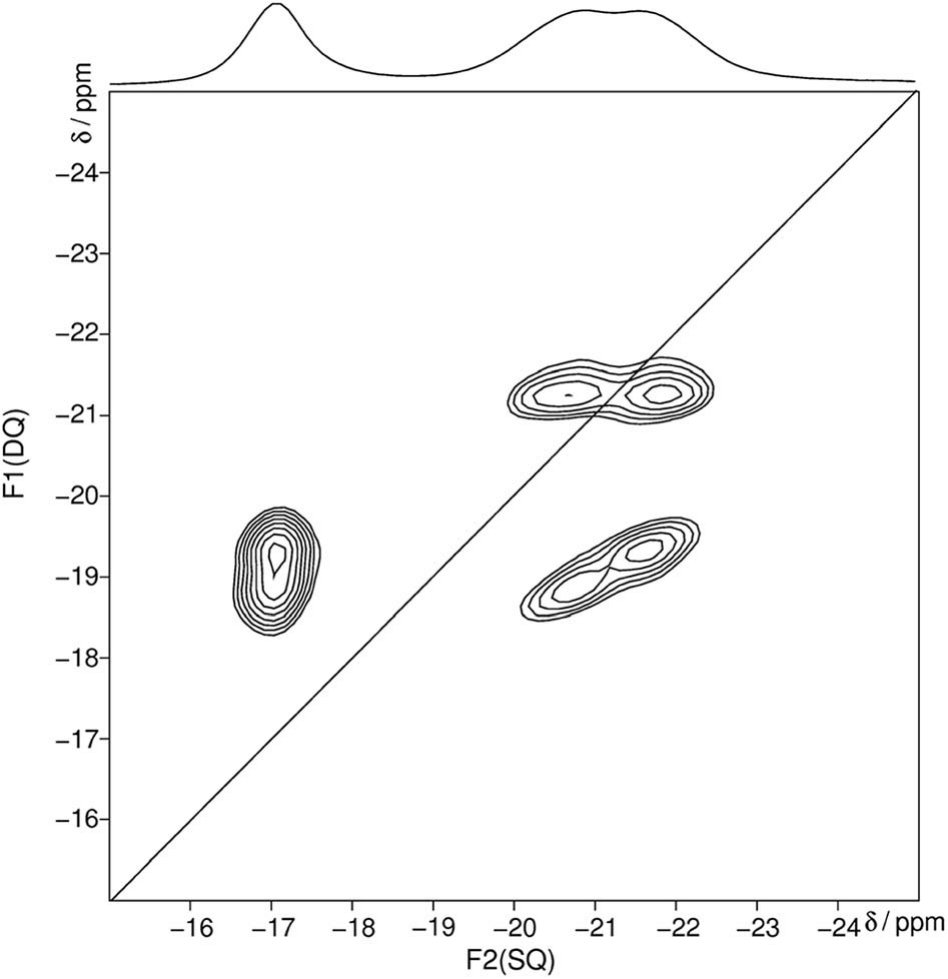
Homonuclear ^31^P–^31^P MAS NMR single-quantum double-quantum correlation spectrum of trismethylammonium cyclotriphosphate [CH_3_NH_3_]_3_P_3_O_9_ obtained at a sample spinning frequency of 12.5 kHz. The 1D projection at the top of the 2D spectrum stems from a separate one-pulse experiment (Fig. S1†). Correlation peaks are shown *via* contour plots. The diagonal line refers to the hypothetic peak position of two isochronous spins (autocorrelation diagonal).

In the following the Q^*n*^ nomenclature is used to describe phosphorus atoms within phosphate tetrahedron units.^57,58^ The variable *n* is defined as the number of bridging oxygen atoms which are connected to the observed phosphorus atom (*n* = 0–3). The homonuclear ^31^P MAS single-quantum (SQ) double-quantum (DQ) correlation spectrum (Fig. 2) shows that all three signals belong to the same crystalline phase because of the presence of three sets of DQ correlation peaks.

The obtained ^31^P isotropic chemical shift values *δ*_iso_, peak areas *A*, spin–lattice relaxation times *T*_1_ and ^31^P anisotropic chemical shift values *δ*_aniso_ are shown in Table 2. These values as well as the correlation pattern are consistent with that of the published structure of the cyclotriphosphate.

**Table 2:** Experimental ^31^P NMR data for trismethylammonium cyclotriphosphate [CH_3_NH_3_]_3_P_3_O_9_; isotropic chemical shift *δ*_iso_, normalized peak area *A*, spin–lattice relaxation time *T*_1_, principal values *δ*_11_, *δ*_22_, *δ*_33_, asymmetry parameter *η* and anisotropic chemical shift *δ*_aniso_ (Fig. S5)

	Peak 1	Peak 2	Peak 3
*δ*_iso_/ppm	−17.1	−20.7	−21.7
*δ*_aniso_/ppm	−152	−162	−159
*η*	0.33	0.26	0.43
*δ*_11_/ppm	50.1	47.5	53.9
*δ*_22_/ppm	16.7	19.4	8.4
*δ*_33_/ppm	−118.1	−129.0	−127.4
*A*/a.u.	1.00	1.08	1.16
*T*_1_/s	28	29	28

**Fig. 3:**
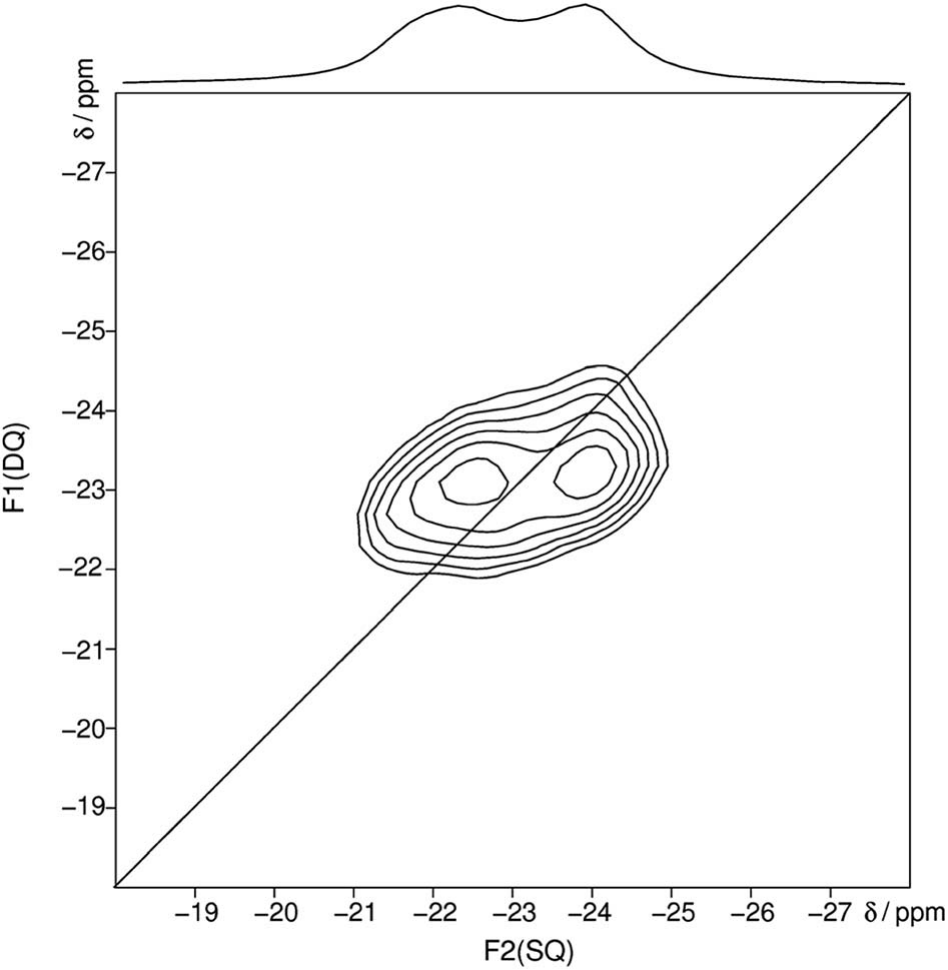
Homonuclear ^31^P–^31^P MAS NMR single-quantum double-quantum correlation spectrum of methylammonium *catena*-polyphosphate [CH_3_NH_3_]PO_3_ received at a sample spinning frequency of 12.5 kHz. The 1D projection at the top of the 2D spectrum stems from a separate one-pulse experiment (Fig. S2†). Correlation peaks are shown *via* contour plots. The diagonal line refers to the hypothetic peak position of two isochronous spins (autocorrelation diagonal).

After heating trismethylammonium cyclotriphosphate slightly above the melting point and subsequent slow cooling another crystalline phase was obtained. The structure of this phase could be characterized by X-ray diffraction and NMR spectroscopy. It was possible to solve and refine the structure from powder X-ray diffraction data by using constraints obtained by NMR spectroscopy. The homonuclear ^31^P MAS single-quantum (SQ) double-quantum (DQ) correlation spectrum (Fig. 3) indicates that these two signals must belong to the same crystalline phase because of their correlation peaks. The connectivity corresponding to the 2D spectrum is consistent with that of a *catena*-polyphosphate with a phosphate chain, which contains two different crystallographic orbits for the phosphorus atoms.

The received ^31^P isotropic chemical shift values *δ*_iso_, peak areas *A*, spin–lattice relaxation times *T*_1_ and ^31^P anisotropic chemical shift values *δ*_aniso_ are shown in Table 3. A minor amorphous side phase can be observed at −12 ppm which differs clearly in *T*_1_ relaxation time (9 s) and full width half maximum from peak 1 and 2 (Fig. S2†). ^31^P NMR gives evidence of two P-sites with equal frequency. The chemical shift anisotropy is typical for Q^2^ phosphates.

**Table 3:** Experimental ^31^P NMR data for methylammonium *catena*-polyphosphate; legend see Table 2; spectrum (Fig. S6)

	Peak 1	Peak 2
*δ*_iso_/ppm	−22.2	−23.8
*δ*_aniso_/ppm	−141	−143
*η*	0.43	0.59
*δ*_11_/ppm	45.0	52.0
*δ*_22_/ppm	4.6	−4.3
*δ*_33_/ppm	−116.2	−119.1
*A*/a.u.	2.09	1.92
*T*_1_/s	48	50

**Fig. 4:**
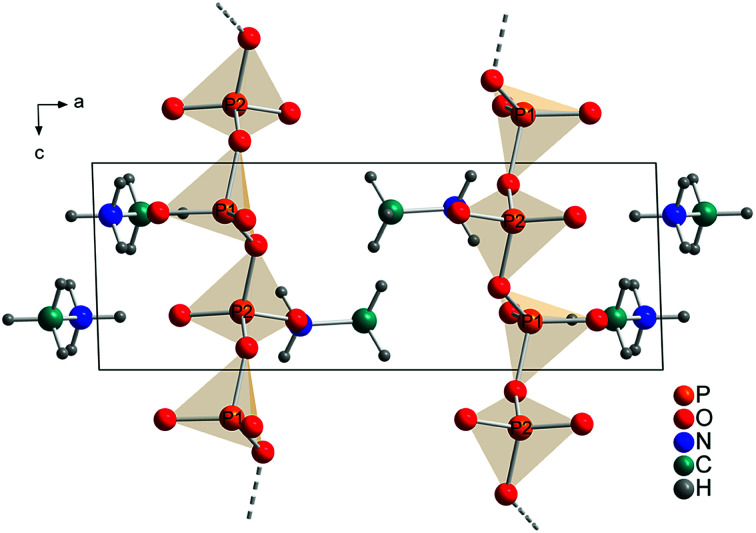
Refined crystal structure of methylammonium *catena*-polyphosphate [CH_3_NH_3_]PO_3_ viewed along [010]. Orange spheres: phosphorus, red spheres: oxygen, blue spheres: nitrogen, cyan spheres: carbon, gray spheres: hydrogen.

The technical process of how to obtain the crystal structure is described in the Experimental part. All observed reflections were indexed with one crystalline phase on the basis of triclinic unit cell. A Rietveld refinement was then performed in space group *P*1̄ with a structure model that contained 2 phosphorus, 6 oxygen, 2 nitrogen, 2 carbon and 12 hydrogen atoms in the asymmetric unit (Fig. 4). This solution is in agreement with the results from XRD, NMR and quantum chemical calculations.

Each P-atom (Q^2^) is connected *via* 2 bridging O-atoms to the neighboring P-atom through the whole structure. The methylammonium molecules are located in the empty space between this polyphosphate chains. The orientation of the methylammonium molecules is influenced by hydrogen bonds between hydrogen atoms attached to nitrogen and non-bridging oxygen atoms of the phosphate chains. For atom N1 two moderate and three weak hydrogen bonds (Fig. S9/S10 and Tables S4/S5†) and for N2 three moderate hydrogen bonds can be observed (Fig. S11/S12 and Tables S4/S5†).^59,60^ On the contrary the orientation of the hydrogen atoms attached to the carbon atom is dominated by intramolecular interactions (staggered conformation). In comparison the hydrogen bond distances are shorter for the calculated than for the experimental structure. This can be explained with the relatively short constrained bond distance for N–H within the experimental structure. Relevant bond distances for hydrogen bonding are given in Tables S4 and S5.† Bridging P–O–P bonds show bigger P–O distances than terminal P–O bonds, as expected. The lengths of the bridging P–O–P bonds are between 1.60(1) and 1.64(2) Å, while the terminal P–O bonds vary between 1.47(1) and 1.50(1) Å. The O–P–O angles vary between 97.9(7) and 129.0(5)° which also represent reasonable values. The arrangement of the phosphate tetrahedron within the phosphate chains shows analogy with (KPO_3_)_*n*_.^61^

**Fig. 5:**
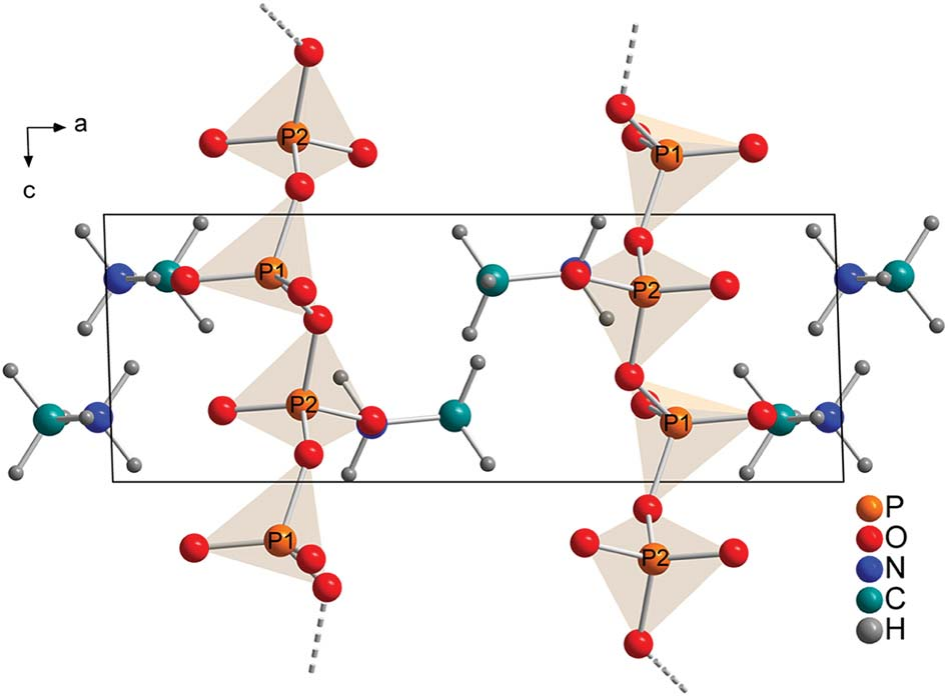
Calculated (5 × 5 × 5 *k*-points, PBE/VdW-DF) crystal structure of methylammonium *catena*-polyphosphate [CH_3_NH_3_]PO_3_ viewed along [010]. Orange spheres: phosphorus, red spheres: oxygen, blue spheres: nitrogen, cyan spheres: carbon, gray spheres: hydrogen.

The comparison of the calculated (Fig. 5) and the refined structure (Fig. 4) shows only minor deviations for bond angles and lengths within the phosphate chains and for the orientations of the methylammonium molecules. Fractional coordinates and selected bond distances are given in Tables S2 and S3.† Similarly the diffraction pattern of the measured and the calculated structures show only minor differences (Fig. S3†).

A comparison of crystalline chain-phosphates of the alkali metals shows an increase of the coordination number as determined with the help of the Voronoi polyhedra of the cations from 7–8 for LiPO_3_ (ICSD collection code 51630) to 8–12 RbPO_3_ (ICSD collection codes 74736, 70035). The newly found crystal structure of [CH_3_NH_3_]PO_3_ fits into this pattern, which is also known as Pauling's first rule, with a coordination number of 11–12.

If crystalline trismethylammonium cyclotriphosphate is molten together with P_4_O_10_ and subsequently quenched an X-ray amorphous compound is obtained. The X-ray powder diffraction pattern (Fig. S4†) shows only 2 broad reflexes in the low angle regime which is consistent with the presence of a glass.

**Fig. 6:**
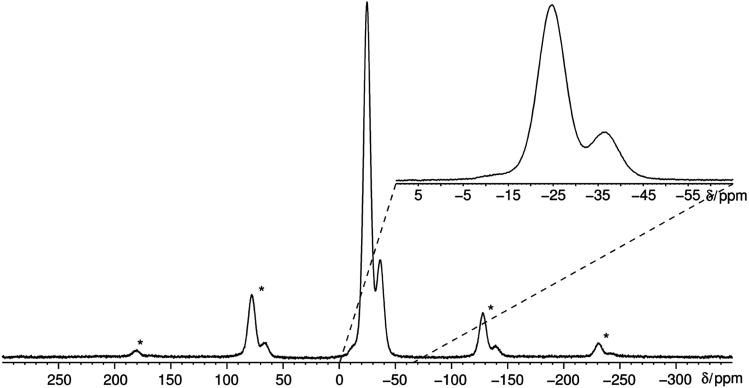
Quantitative ^31^P MAS NMR spectrum of methylammonium phosphate glass of the composition 3.11 [CH_3_NH_3_]_3_P_3_O_9_·P_4_O_10_ measured at a sample spinning frequency of 12.5 kHz. The spectrum shows three signals corresponding to three different crystallographic orbits of phosphorus atoms. The signals appear at −12.0, −24.7 and −36.7 ppm. The signal at −12.0 ppm is negligible due to its very low peak area of 1% relative to peak 2. The spectrum includes all rotational side-bands signed with an asterisk.

**Fig. 7:**
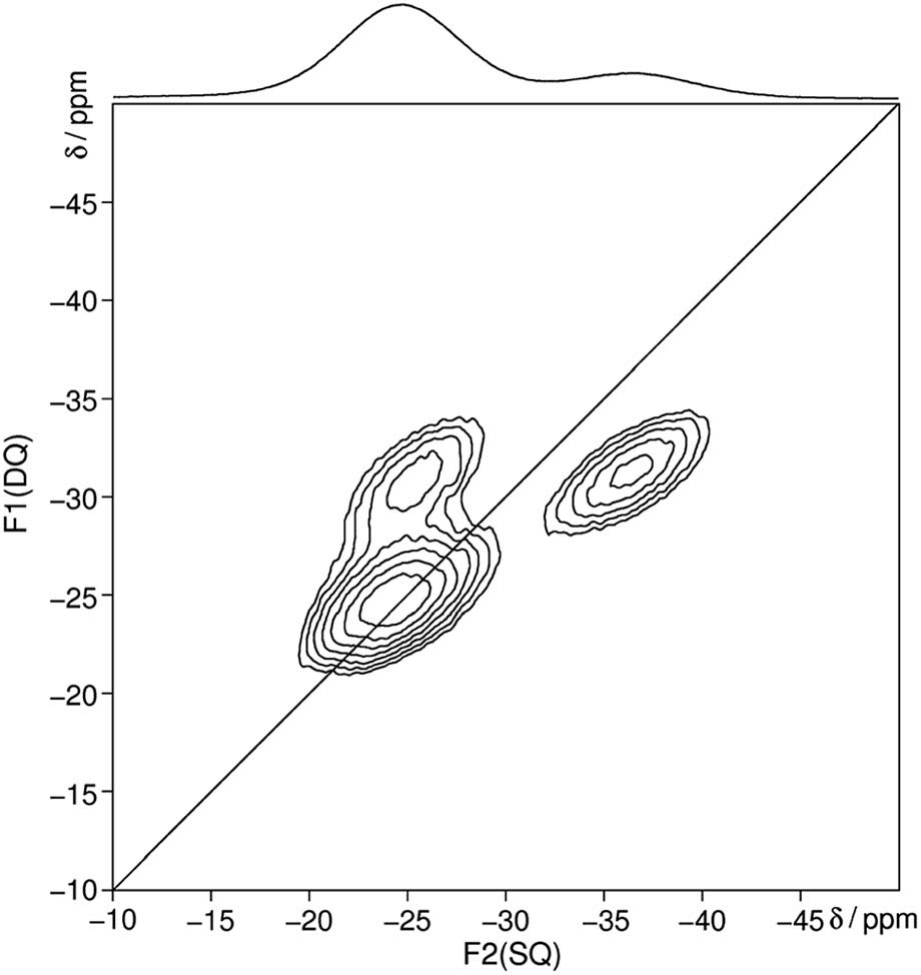
Homonuclear ^31^P–^31^P MAS NMR single-quantum double-quantum correlation spectrum of methylammonium phosphate glass of the composition 3.11 [CH_3_NH_3_]_3_P_3_O_9_·P_4_O_10_ recorded at a sample spinning frequency of 12.5 kHz. The 1D projection at the top of the 2D spectrum stems from a separate one-pulse experiment (Fig. 6). Correlation peaks are shown *via* contour plots. The diagonal line refers to the hypothetic peak position of two isochronous spins (autocorrelation diagonal).

The ^31^P MAS NMR spectrum (Fig. 6) shows a signal at −24.7 ppm which can be assigned to a Q^2^ phosphate and a signal at −36.7 ppm which can be assigned to a Q^3^ phosphate. The full width half maximum of the observed peaks is relatively broad (850 Hz) which is consistent with the presence of a glassy phosphate which consists mainly out of Q^2^ and Q^3^ phosphate units (peak areas Q^2^ : Q^3^ = 5 : 1). Note that there is no signal at −45 ppm which means that P_4_O_10_ reacts quantitatively. The obtained ^31^P isotropic chemical shift values *δ*_iso_, peak areas *A*, spin–lattice relaxation times *T*_1_ and ^31^P anisotropic chemical shift values *δ*_aniso_ are shown in Table 4. The homonuclear ^31^P MAS single-quantum (SQ) double-quantum (DQ) correlation spectrum (Fig. 7) indicates that these two signals must belong to the same amorphous phase because of their correlation peaks. This correlation pattern as well as the peak areas are consistent with that of a polyphosphate which contains cross-linked phosphate chains. The lower the P_4_O_10_ content the lower the amount of cross-links between chains, which means the glass structure of pure trismethylammonium cyclotriphosphate should consist mostly of long chains as expected from its crystalline approximant,^62,63^*i.e.* [CH_3_NH_3_]PO_3_.

**Table 4:** Experimental ^31^P NMR data for methylammonium phosphate glass; legend see Table 2; spectrum (Fig. S7)

	Peak 1	Peak 2
*δ*_iso_/ppm	−24.7	−36.7
*δ*_aniso_/ppm	−147	−133
*η*	0.40	0.16
*δ*_11_/ppm	43.7	14.9
*δ*_22_/ppm	4.6	0.7
*δ*_33_/ppm	−122.4	−125.6
*A*/a.u.	5.08	1
*T*_1_/s	16	16

**Fig. 8:**
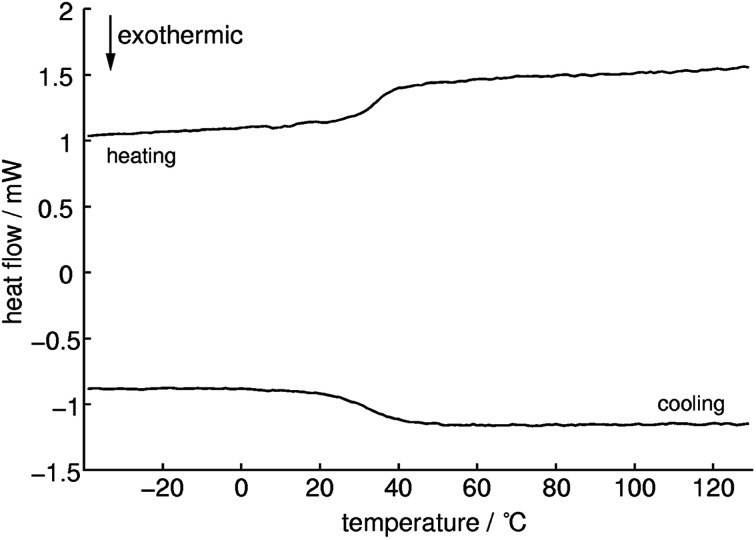
DSC measurement of methylammonium phosphate glass of the composition 3.71 [CH_3_NH_3_]_3_P_3_O_9_·P_4_O_10_ between −40 and 130 °C with a heating/cooling rate of 5 K min^−1^ (heating: top line, cooling: bottom line). Onset temperature of the glass transition *T*_g_ at 33 °C.

Differential scanning calorimetry measurements (Fig. 8) show an endothermic signal with an onset temperature of 33 °C during heating which can be assigned to a glass transition. Whereas cooling approximately at the same temperature an exothermic process occurs which is indicating a reversible process. This could be confirmed with successive measurements which showed almost the same results (1^st^: 32.9, 2^nd^: 32.5 and 3^rd^: 32.9 °C). The *T*_g_ of methylammonium phosphate glass is considerably lower than for CsPO_3_ glass (*T*_g_ = 240 °C).^64^ No signals for cold crystallization and subsequent melting could be observed which means that this compound tends not to crystallize. The quotient of the change in specific heat capacity and the heat capacity of the crystalline phase Δ*C*_P_/*C*_P_(cryst) is 0.4 ± 0.1 which is a relatively low value and therefore it can be expected that a fairly strong glass in the sense of Angell is formed.^65^

**Fig. 9:**
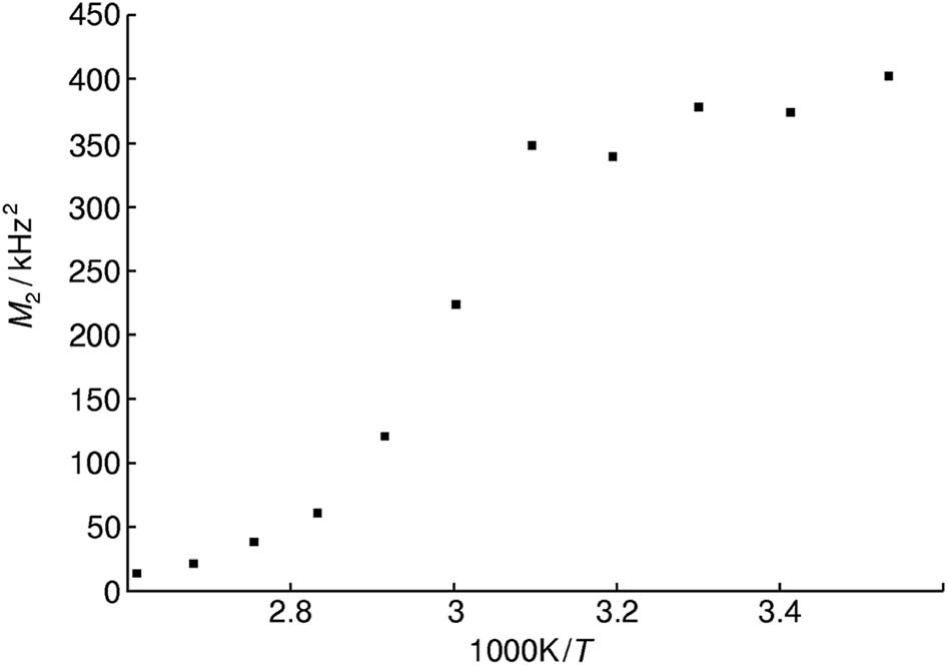
Plot of second moments *M*_2_ of the static ^31^P NMR line shape for methylammonium phosphate glass of the composition 4.14 [CH_3_NH_3_]_3_P_3_O_9_·P_4_O_10_ at various reciprocal temperatures.

Static variable temperature ^31^P-NMR experiments show a sharp decrease of the second moment *M*_2_ at elevated temperatures. This decrease is indicative for an activation of rotational and translational degrees of freedom of the phosphate tetrahedron, which lead to motional averaging like in an isotropic liquid phase, as expected above the glass transition temperature. The activation energy for this process can be estimated by the Waugh–Fedin equation *E*_A_ ≈ 1.617 × 10^−3^*T*_onset_ eV K^−1^ with an error of approximately 10% for *T*_onset_ which results in an activation energy *E*_A_ of 0.52 ± 0.05 eV.^66^ The temperature *T*_onset_ is defined as the onset temperature (323 ± 32 K) for a decrease in the second moment *M*_2_ of the NMR spectrum during heating (Fig. 9).

**Fig. 10:**
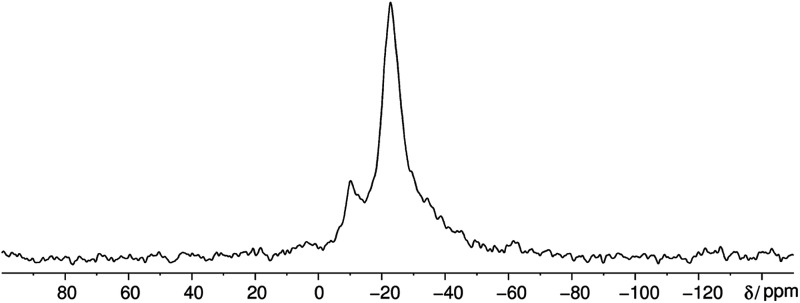
Static ^31^P NMR spectrum of methylammonium phosphate glass of the composition 4.14 [CH_3_NH_3_]_3_P_3_O_9_·P_4_O_10_ obtained at 383 K. The spectrum shows three signals corresponding to three different crystallographic orbits of phosphorus atoms. The signals appear at −10, −23 and −35 ppm.

Interestingly the static ^31^P NMR spectrum obtained at 383 K shows 3 different signals at 383 K at approximately −10, −23 and −36 ppm (Fig. 10). Usually it is not possible to resolve different phosphorus environments with ^31^P NMR at elevated temperatures within phosphate glasses due to fast exchange as for instance in silver phosphate glass systems (unpublished results). Solely in aluminum phosphate glasses this finding is reported in literature where aluminum phosphate subunits are stable on the NMR timescale and lead to resolvable peaks.^67^ The vast majority of the phosphorus sites have a Q^2^ environment which is in agreement with the phase transition of the cyclophosphate into the *catena*-polyphosphate at elevated temperatures.

## 4. Conclusions

We could show that the crystal structure of trismethylammonium cyclotriphosphate undergoes a phase transition from space group *P*2_1_/*n* to *P*1̄. Interestingly during this process the cyclic phosphate transforms into a *catena*-polyphosphate which is the thermodynamical stable phase at higher temperature. If the trismethylammonium cyclotriphosphate is reacted with P_4_O_10_ at elevated temperatures and fast subsequent cooling is applied, a glassy polyphosphate containing Q^2^ and Q^3^ phosphate environments can be obtained. This glass shows a low glass transition temperature *T*_g_ of 33 °C which enables the possibility to incorporate thermal sensitive compounds into the glass melt. Hence this can be especially interesting for embedding organic molecules. To the best of our knowledge this is the first binary phosphate glass system free of acidic protons which has a glass transition temperature below 40 °C. Such glasses are also interesting for fundamental studies about dynamic processes of the α-process of the glass transition in phosphate glasses, because the breaking of P–O–P bridges could be studied *in situ* by NMR.

## Conflicts of interest

There are no conflicts to declare.

## Supplementary Material

RA-009-C8RA07736C-s001
