# Supplementary material for: Synthesis and characterization of methylammonium phosphates as crystalline approximants for anhydrous, low melting phosphate glasses
Source: RSC Adv. 2019 Jan 15;9(4):1822–30. doi: 10.1039/c8ra07736c (PMC9059716; doi:10.1039/c8ra07736c)
Supplement: RA-009-C8RA07736C-s001 [file RA-009-C8RA07736C-s001.pdf]

Electronic Supplementary Material (ESI) for RSC Advances.
This journal is © The Royal Society of Chemistry 2019

Supporting Information

Martin Mangstl,[a] Jan Konrad Wied,[a] Johannes Weber,[a] Christian Pritzel,[b] Reinhard Trettin[b] and Jörn Schmedt auf der Günne*[a]

Carbon monoxide detection:

The reaction of P_4O_{10} and N-methylformamide was carried out for 24 h at 45 °C within a closed Schlenk flask. Afterwards, the generated gas was detected with an electrochemical detector (Figaro TGS5042), which meets EN50191 requirements.

Table S1. Best 10 scores for indexing using a LeBail extraction with a least-squares optimization within the FOX program.

Score	*a*	*b*	*c*	*α*	*β*	*γ*
18.9	13.2145	7.88568	4.65417	91.0995	87.8988	106.557
15.2	13.504	8.01009	7.73902	100.328	102.064	103.652
14.9	13.3206	9.22618	9.08799	118.936	73.6459	104.48
14.6	7.88087	6.65105	4.65183	91.4389	91.0045	108.042
14.5	13.0615	8.31856	8.31356	105.348	74.8388	94.6982
13.1	13.2337	7.89056	4.6815	92.8186	93.58	106.314
11.7	13.2463	7.9111	4.68491	92.8001	93.5532	106.37
11.6	13.1995	7.87323	4.65142	91.201	87.9078	106.574
11.5	13.2085	7.87525	4.65142	91.3966	92.2543	106.454
11.4	13.2357	9.33738	4.65142	118.537	74.0653	102.168

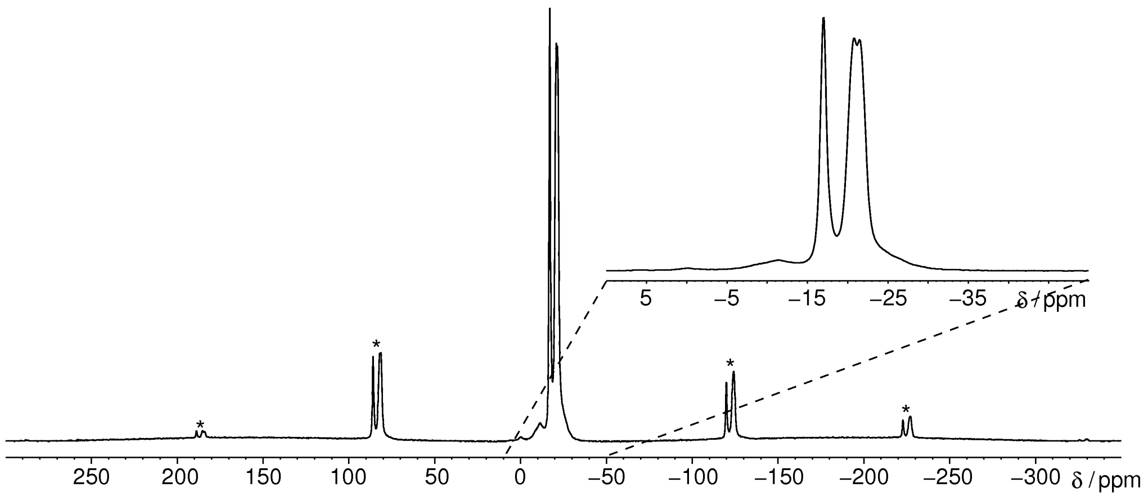

Figure S1. Quantitative ^{31}P MAS NMR spectrum of trismethylammonium cyclotriphosphate obtained at a sample spinning frequency of 12.5 kHz. The spectrum shows three signals corresponding to three different crystallographic orbits of phosphorus atoms within the molecule which appear at −17.1, −20.7 and −21.7 ppm. The signals at 0 and −11.3 ppm are negligible due to its very low intensities and the higher half width full maximum which indicates an amorphous side-phase. The spectrum includes all rotational side-bands signed with an asterisk.

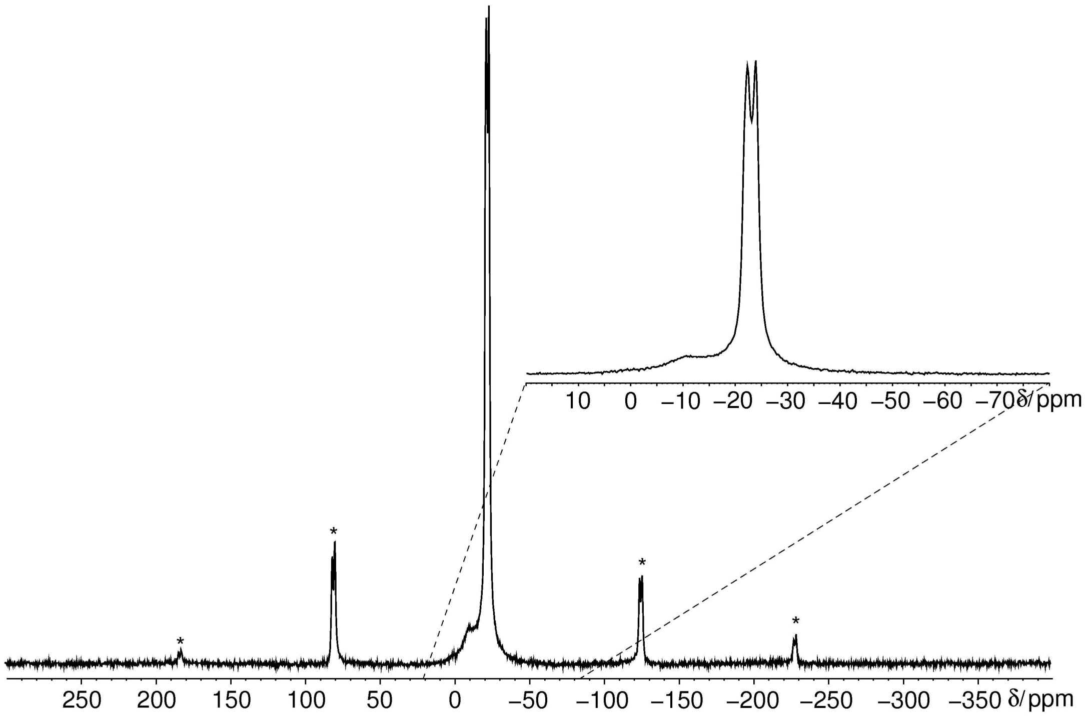

Figure S2. Quantitative ^{31}P MAS NMR spectrum of methylammonium catena-polyphosphate obtained at a sample spinning frequency of 12.5 kHz. The spectrum shows three signals corresponding to three different crystallographic orbits of phosphorus atoms. The main signals appear at −22.2 and −23.8 ppm. A small amount of an amorphous side-phase causes a signal (higher full width half maximum) at −16.0 ppm. The spectrum includes all rotational side-bands signed with an asterisk.

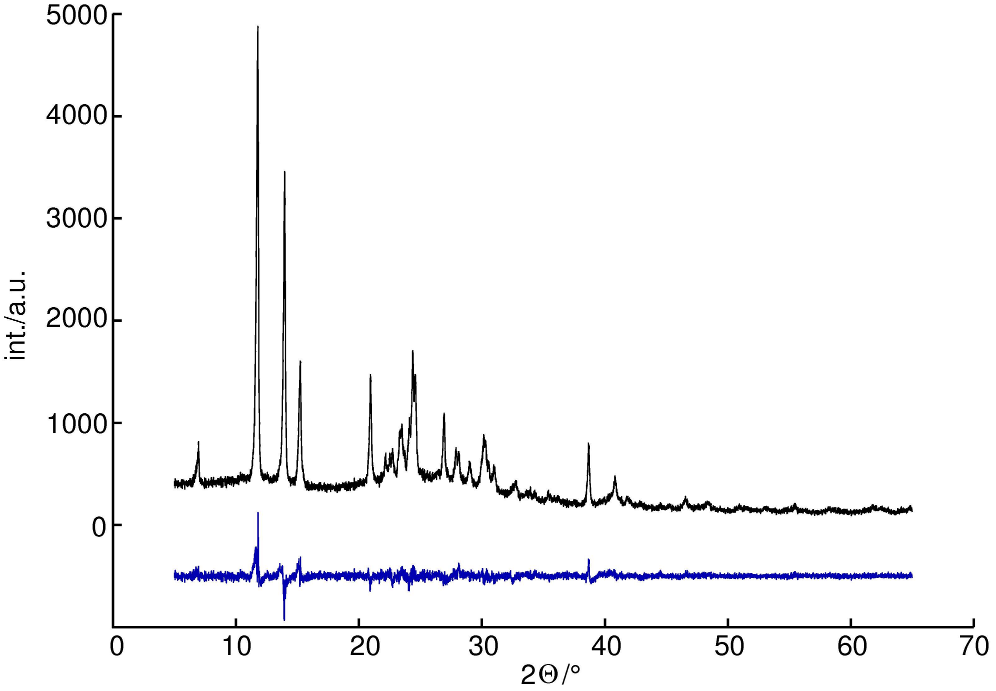

Figure S3. Calculated (5x5x5 *k*-points, PBE/VdW-DF) powder diffraction pattern (black line) of methylammonium catena-polyphosphate, as well as the difference profile (blue line) of the measured (Figure 1) and calculated diffraction pattern (χ^2 = 1.323, R_p = 0.054).

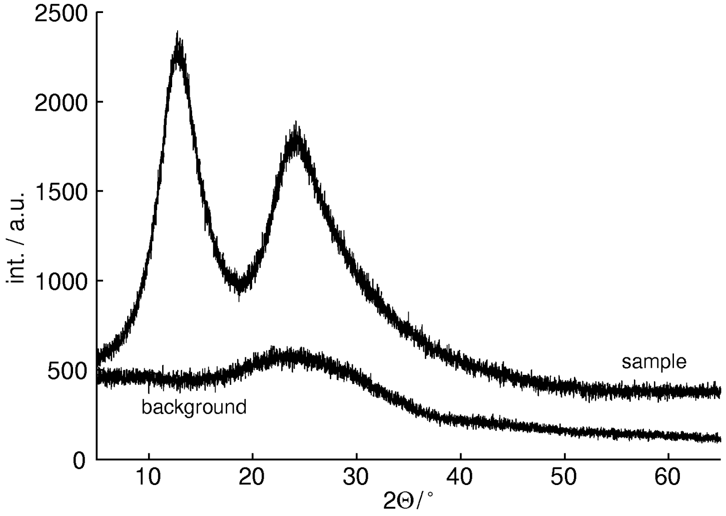

Figure S4. Observed powder diffraction pattern of methylammonium phosphate glass measured with Cu$K_{\alpha 1}$ radiation (top), as well as the background of the capillary (bottom).

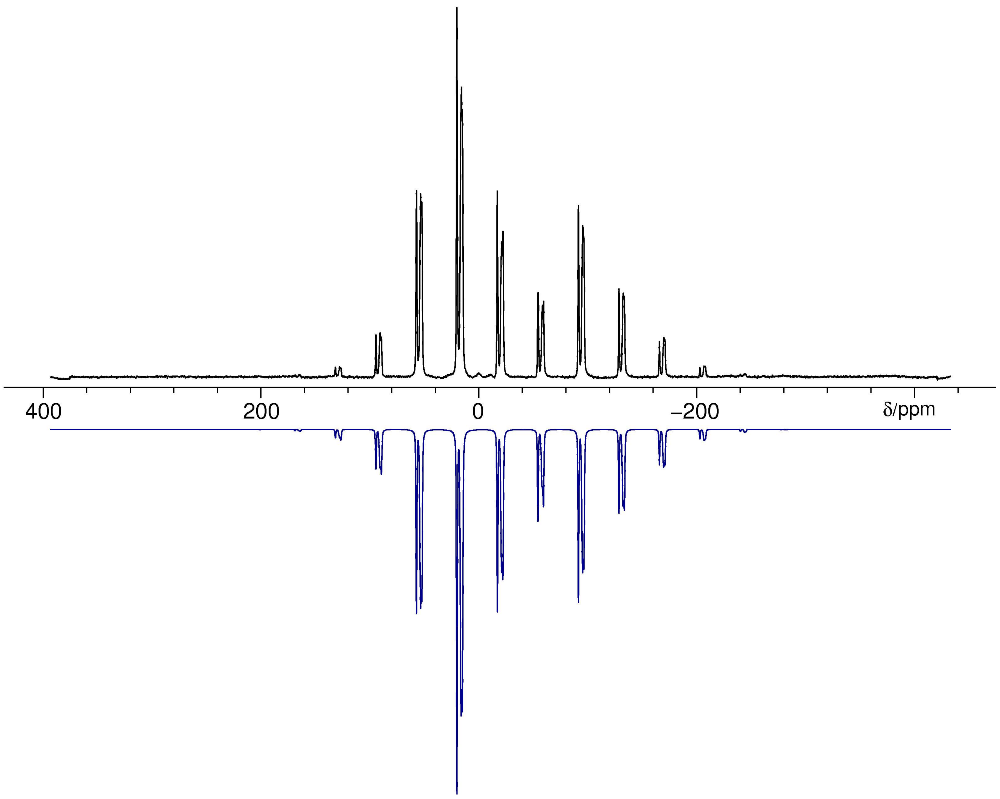

Figure S5. Experimental (black line) and simulated (blue line) ^{31}P MAS NMR spectrum of trismethylammonium cyclotriphosphate obtained at a sample spinning frequency of 5 kHz.

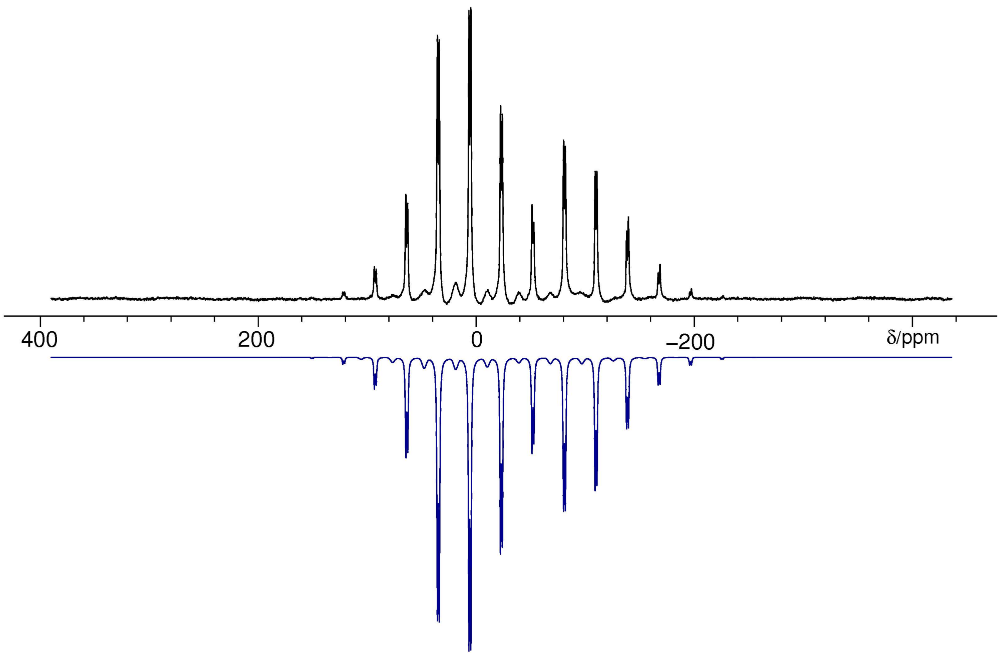

Figure S6. Experimental (black line) and simulated (blue line) ^{31}P MAS NMR spectrum of methylammonium catena-polyphosphate obtained at a sample spinning frequency of 3.5 kHz.

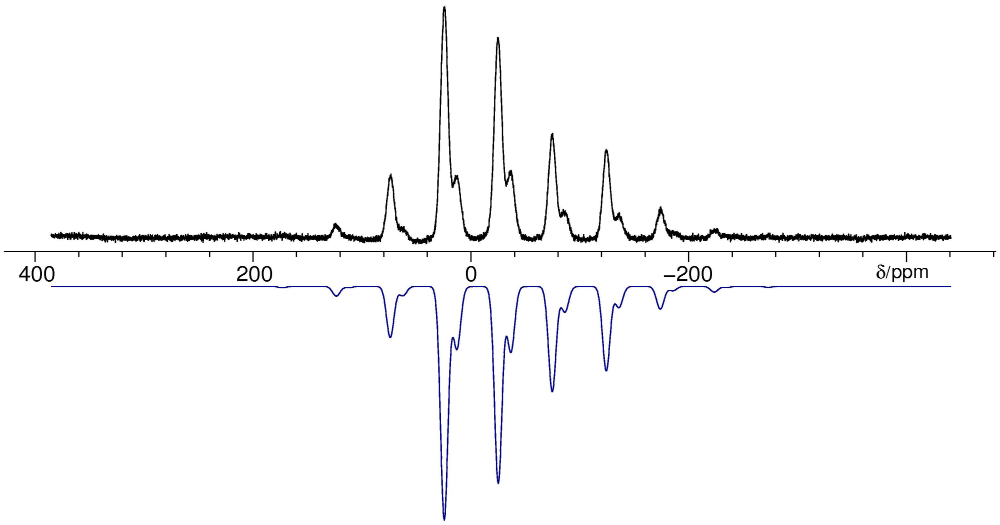

Figure S7. Experimental (black line) and simulated (blue line) ^{31}P MAS NMR spectrum of methylammonium phosphate glass obtained at a sample spinning frequency of 6 kHz.

Table S2. Fractional coordinates of the atoms in the unit cell of methylammoniumcatenapolyphosphate by Rietvelt refinement in comparison with the calculated one (5x5x5 *k*-points, PBE/VdW-DF).

Atom	x_{exp}	x_{exp}	z_{exp}	x_{calc}	y_{calc}	z_{calc}
P1	0.76743(39)	0.17049(62)	0.7665(12)	0.77519	0.15756	0.78002
P2	0.74326(40)	0.37623(67)	0.2814(12)	0.74190	0.36797	0.29139
O1	0.88596(57)	0.2352(18)	0.7594(36)	0.89242	0.22427	0.75062
O2	0.7327(11)	-0.02504(99)	0.7241(35)	0.71910	0.96793	0.70959
O3	0.71428(88)	0.2971(11)	0.6017(15)	0.71895	0.28539	0.61102
O4	0.85320(67)	0.4915(18)	0.2627(39)	0.85400	0.48447	0.26427
O5	0.7368(11)	0.1965(10)	0.1054(14)	0.73213	0.18595	0.10628
O6	0.64728(56)	0.4299(13)	0.2401(34)	0.65305	0.44132	0.22812
N1	0.97066	0.65321	0.74714	0.95347	0.64963	0.76107
N2	0.35925	0.21525	0.78115	0.36396	0.21732	0.78433
C1	0.91678	0.79275	0.75301	0.90736	0.80264	0.77223
C2	0.47298	0.22332	0.76300	0.47795	0.21978	0.74456
H1	0.95155	0.58702	0.90357	0.92483	0.57119	0.93937
H2	1.04020	0.70235	0.74309	0.03422	0.68777	0.76076
H3	0.93740	0.86414	0.58428	0.93792	0.88802	0.58969
H4	0.84178	0.73974	0.75738	0.82218	0.75077	0.76105
H5	0.93603	0.86540	0.92119	0.92786	0.87466	0.97315
H6	0.95282	0.58586	0.59123	0.92894	0.57647	0.57615
H7	0.35280	0.32441	0.79270	0.35594	0.34450	0.78013
H8	0.32903	0.15506	0.93662	0.33297	0.15831	0.97819
H9	0.47994	0.10558	0.75054	0.48243	0.08397	0.74858
H10	0.50558	0.28825	0.59530	0.50819	0.27969	0.53865
H11	0.50688	0.28133	0.93143	0.52328	0.29656	0.91711
H12	0.32782	0.16147	0.62499	0.32147	0.14346	0.61871

Table S3. Selected bond distances from PXRD, d_{exp}, and from quantum chemical calculations, d_{exp}.

Atomic pair	Atomic distances in Å d_{exp}	d_{calc}	Atomic pair	Atomic distances in Å d_{exp}	d_{calc}
P1-O1	1.505(11)	1.490	P1-O2	1.491(9)	1.505
P1-O3	1.597(11)	1.641	P1-O5	1.638(9)	1.636
P2-O3	1.617(9)	1.619	P2-O4	1.479(14)	1.508
P2-O5	1.607(9)	1.637	P2-O6	1.468(12)	1.491
N1-C1	1.472	1.499	N2-C2	1.486	1.506
N1-H1	0.89	1.04	N2-H7	0.89	1.04
N1-H2	0.89	1.02	N2-H8	0.89	1.04
N1-H6	0.89	1.04	N2-H12	0.89	1.04
C1-H3	0.96	1.09	C2-H9	0.96	1.09
C1-H4	0.96	1.09	C2-H10	0.96	1.09
C1-H5	0.96	1.09	C2-H11	0.96	1.09
O2-H8	1.92	1.78	O2-H12	2.00	1.77
O4-H1	2.09	1.79	O4-H2	2.36	1.27
O4-H6	2.04	1.81	O6-H7	1.95	1.73
O6-H10	2.48	2.42	O6-H11	2.41	2.31

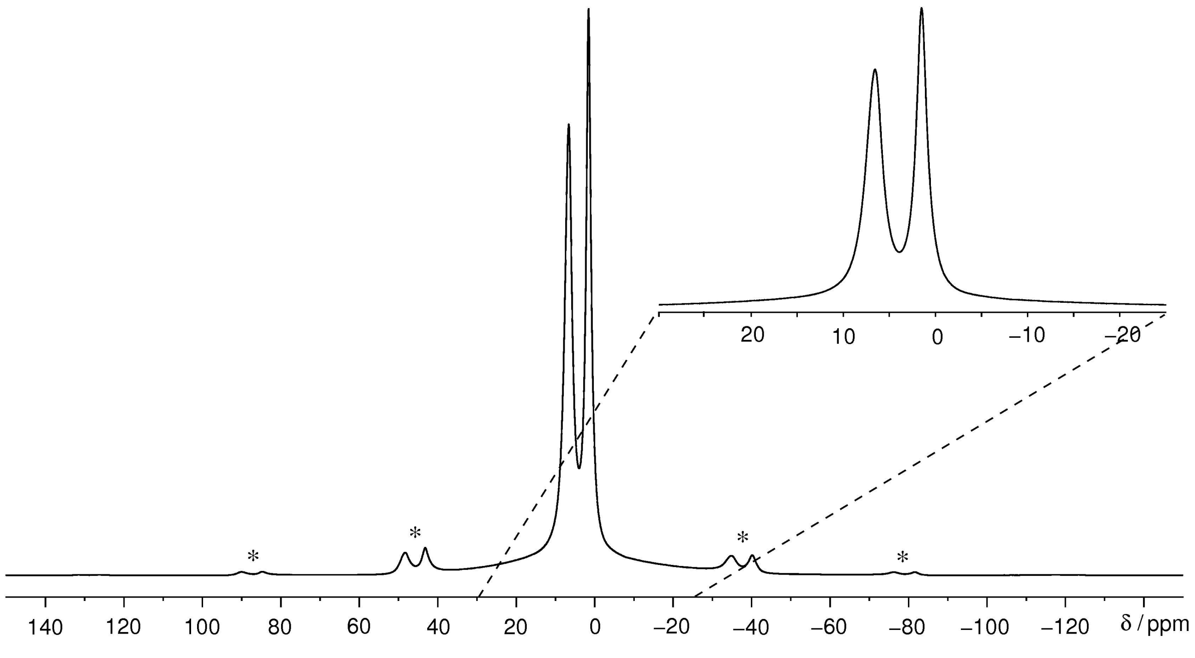

Figure S8. Quantitative ^1H MAS NMR spectrum of methylammonium catena-polyphosphate obtained at a sample spinning frequency of 25 kHz. The spectrum shows two signals corresponding to 2 different environments of hydrogen atoms, i.e. the H-atoms of the CH_3 and NH_3 functions, respectively. The signals appear at 6.6 (N-H) and 1.5 ppm (C-H) and show almost identical peak areas. The spectrum includes all rotational side-bands denoted with an asterisk.

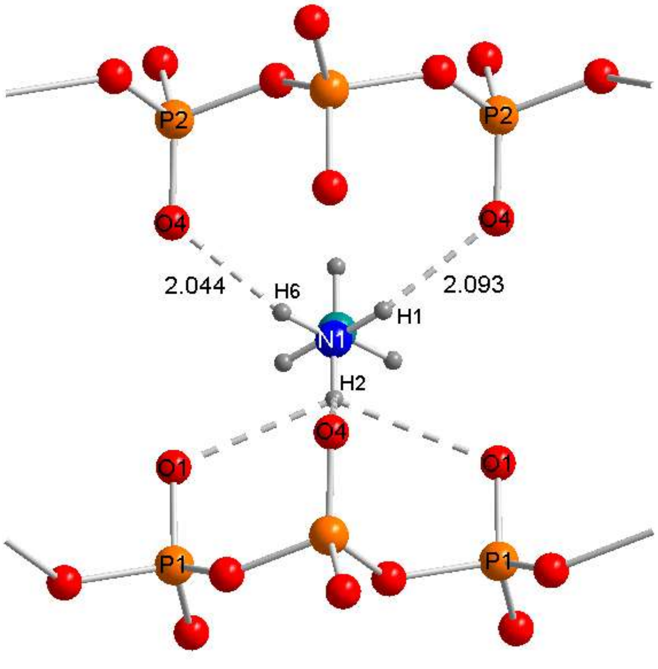

Figure S9. Graphical visualization of medium hydrogen bonds in Ångström achieved by powder XRD structure refinement.

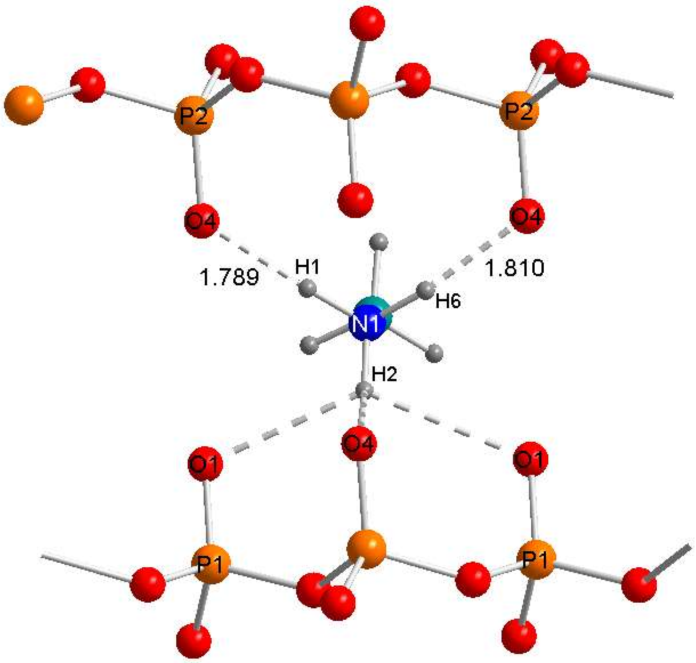

Figure S10. Graphical visualization of medium hydrogen bonds in Ångström achieved by quantum chemical calculations (PBE/VdW-DF).

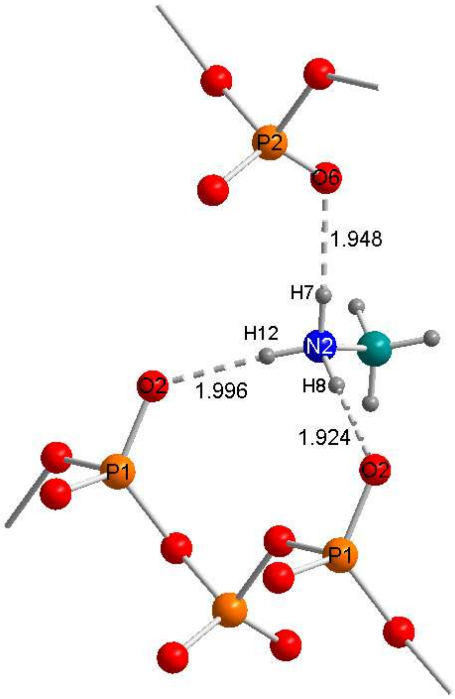

Figure S11. Graphical visualization of medium hydrogen bonds in Ångström achieved by powder XRD structure refinement.

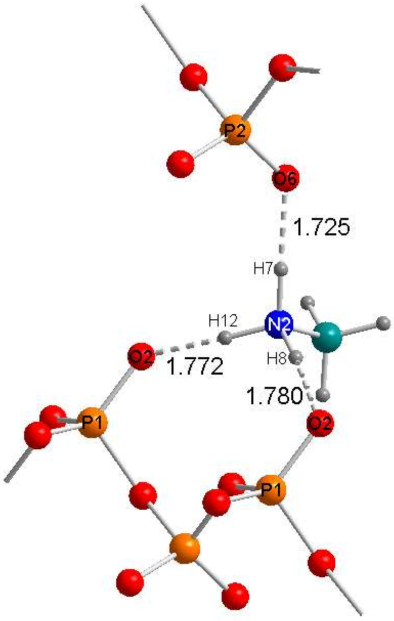

Figure S12. Graphical visualization of medium hydrogen bonds in Ångström achieved by quantum chemical calculations (PBE/VdW-DF).

Table S4. Relevant experimental bond distances *d* in Ångström and angles *ω* in degree for hydrogen bonding.

Atoms	d_{exp}(N–O) / Å	d_{exp}(N–H) / Å	d_{exp}(O–H) / Å	ω_{exp}(N-H-O) / °
N2-H7-O6	2.83(1)	0.89	1.95(1)	170.6(3)
N2-H12-O2	2.88(1)	0.89	2.00(1)	174.1(3)
N2-H8-O2	2.81(1)	0.89	1.92(1)	178.5(3)
N1-H1-O4	2.92(2)	0.89	2.09(2)	153.5(4)
N1-H6-O4	2.86(2)	0.89	2.04(2)	151.0(4)
N1-H2-O1	2.99(1)	0.89	2.53(2)	112.7(2)
N1-H2-O4	2.87(1)	0.89	2.36(1)	116.8(2)
N1-H2-O1	2.95(1)	0.89	2.50(2)	111.9(2)

Table S5. Relevant theoretical bond distances *d* in Ångström and angles *ω* in degree for hydrogen bonding (PBE/VdW-DF).

Atoms	d_{qalc}(N–O) / Å	d_{qalc}(N–H) / Å	d_{qalc}(O–H) / Å	ω_{qalc}(N-H-O) / °
N2-H7-O6	2.77	1.04	1.73	178.2
N2-H12-O2	2.79	1.04	1.77	165.4
N2-H8-O2	2.81	1.04	1.78	172.9
N1-H1-O4	2.80	1.04	1.79	162.6
N1-H6-O4	2.81	1.04	1.81	161.8
N1-H2-O1	3.07	1.02	2.51	113.5
N1-H2-O4	3.02	1.02	2.27	128.6
N1-H2-O1	3.06	1.02	2.57	109.4
